# Comparison of Short- and Medium-Term Clinical Outcomes between Transradial Approach and Transfemoral Approach in a High-Volume PCI Heart Center in China

**DOI:** 10.1371/journal.pone.0118491

**Published:** 2015-03-31

**Authors:** Peiyuan He, Yuejin Yang, Shubin Qiao, Bo Xu, Min Yao, Yongjian Wu, Jinqing Yuan, Jue Chen, Haibo Liu, Jun Dai, Xiao Yang, Xinran Tang, Yang Wang, Wei Li, Runlin Gao

**Affiliations:** 1 Department of Cardiology, Cardiovascular Institute and Fuwai Hospital, National Center for Cardiovascular Diseases China, Chinese Academy of Medical Sciences and Peking Union Medical College, Beijing, 100037, China; 2 Medical Research & Biometrics Center, Cardiovascular Institute and Fuwai Hospital, National Center for Cardiovascular Diseases China, Chinese Academy of Medical Sciences and Peking Union Medical College, Beijing, 100037, China; Azienda Ospedaliero-Universitaria Careggi, ITALY

## Abstract

**Background:**

Transradial approach (TRA) outweighed transfemoral approach (TFA) in acute coronary syndrome patients because the former has better short-term outcomes in high-volume percutaneous coronary intervention (PCI) centers. Our study was one of the limited studies specifically in comparing the short- and medium-term effects of TRA and those of TFA in patients undergoing elective PCIs.

**Methods:**

A total of 21,242 patients who underwent elective PCI with stent implantation were included. Using propensity score methodology, 1,634 patient pairs were matched. Major clinical outcomes and PCI-related complications between TRA and TFA were compared.

**Results:**

In the propensity score-matched patients, the rates of in-hospital net adverse clinical events, which included death, myocardial infarction (MI), target vessel revascularization (TVR), stroke, and major bleeding, were much lower with TRA than with TFA (1.8% vs. 3.9%, *P* < 0.001). This difference was mainly due to the lower rate of major bleeding (0.6% vs. 1.8%, *P* < 0.001) and the decreased rate of MI (1.1% vs. 1.9%, *P* = 0.060). PCI-related dissection and thrombosis were similar between the TRA and TFA groups (both *P* > 0.05). Meanwhile, one-year incidence rates of major adverse cardiovascular events, which included death, MI, and TVR, were also similar (4.1% vs. 4.9%, *P* = 0.272) in TRA and TFA. Multivariable regression analyses showed that TRA was an independent predictor of the low rate of in-hospital net adverse clinical events (odds ratio, 0.53; 95% confidence interval, 0.40 to 0.71), but not of major adverse cardiovascular events at one-year follow-up (hazard ratio, 1.01; 95% confidence interval, 0.96 to 1.06).

**Conclusions:**

In patients undergoing elective PCI, TRA patients had lower rates of in-hospital net adverse clinical outcomes compared with TFA patients. TRA might be recommended as a routine approach in high-volume PCI hospitals for elective PCIs.

## Introduction

It has been 20 years since the first percutaneous coronary intervention (PCI) through transradial approach (TRA) was successfully performed by Dr. Kiemeneij and Laarman.[1] Since then, this method has been increasingly adopted because of its superior features (lower vascular complications, shorter hospitalization, and better experience for patients) compared with the transfemoral approach (TFA).[2–5] The application rate of TRA varies across countries. In Asia and some European countries, TRA is a routine approach. By contrast, the application rate of TRA is very low in the US.[6,7] China is among the first countries to use the TRA strategy and to maintain its practice. A cross-national survey in China showed that TRA accounted for 56.3% of all routes for PCI in 2007, and this percentage increased to 76.1% in 2011.[8] In Fuwai hospital, TRA has been used since 2000. More than 10 000 PCIs are performed each year, and TRA has accounted for approximately 90% of all routes. Learning curves exist for TRA beginners, however, most of the interventionists are systematically trained and have accumulated a great amount of experience in implementing TRA.[9] The RIVAL trial (trial in RadIal Vs. femorAL access for coronary intervention) suggested that institution with high volume of PCI had better 30 d outcomes with TRA compared with TFA in acute coronary syndrome patients.[10] While very few studies have tested that in patients undergoing elective PCI. Thus, a comparison of major procedural and clinical outcomes between TRA and TFA during hospitalization and at one-year follow-up was performed. Meanwhile, a propensity score methodology was used to decrease the disparities.

## Material and Methods

### Patient selection and data collection

**Fig 1:**
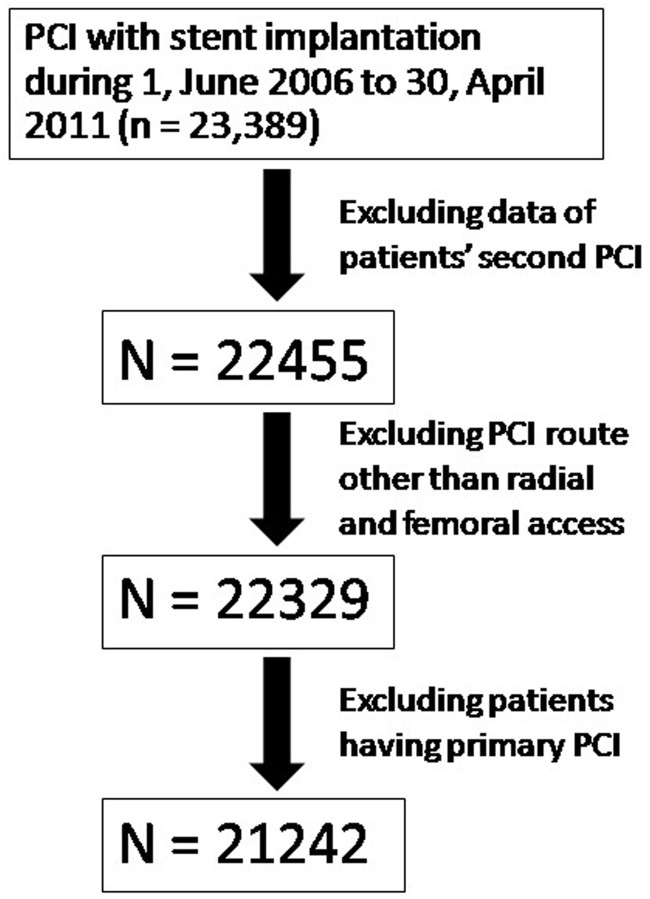
The flow chart of patient selection.

This study was conducted in a single institution—Fuwai hospital, the largest heart center in China. The annual amount of PCI in Fuwai hospital was more than 10,000 after year 2011, and the proportion that uses TRA in PCI is increasing. The statistical method of the study was based on a post-hoc analysis of a prospective database. From 1 June 2006 to 30 April 2011, a total of 23,389 patients who have undergone PCI with stent implantation were included in the study. The flow chart for patient selection was shown in Fig 1. Finally, 21,242 patients were included in the analysis. The angiographic data was downloaded from the digital database of the catheter laboratory. The baseline data and the in-hospital outcomes were extracted from the medical charts. A group of systematically trained medical students worked on the data extraction. Four senior fellows constituted the quality control committee, in which one person was in charge of adjudicating ambiguous endpoints and the others were responsible for data inspection. Informed consents were obtained upon patient admission, and follow-up checkups were performed at 6 months and 1 year after discharge. The protocol of this study was approved by the Ethics Committee of Fuwai hospital, and patient information was anonymized and de-identified prior to analysis.

### The procedure

TRA is preferred in our institution. Under certain conditions however, doctors are still willing to choose the femoral route. Such conditions included a failed Allen’s test, a weak or non-palpable pulse, and a history of coronary artery bypass surgery. Doctors had good expertise both with TRA and TFA. 36 interventionists performed 19,363 procedures (with available name of the operator in the medical record) during approximately 5 years. Among them, when 13 operators who performed less than 15 procedures were excluded, then each operator performed about 150 procedures per year. Using that as benchmark, 14 operators had procedures less than 150 annually; 4 operators had procedures between 150 to 300 annually; and 5 operators had procedures more than 300 annually.

At the start of the procedure, 1 ml 1% lidocaine was subcutaneously injected for local anesthesia, and the puncturing needle and the sheath were used to build the route. The hemostasis method was easier for TRA than for TFA. In TRA patients, hemostasis was achieved by manual compression of the puncturing site, followed by clamp placement over the artery. In TFA patients, sandbag compression was demanded after manual compression, and patients were required to stay in bed for hours before ambulation.

### Endpoint definition and follow-up checkups

The primary endpoint was defined as in-hospital net adverse clinical events (NACE), which included all causes of death, myocardial infarction (MI), target vessel revascularization (TVR), stroke, and major bleeding. The secondary endpoint was defined as major adverse cardiovascular events (MACE) at one-year follow-up, which included all causes of death, MI, and TVR. Death was categorized as either cardiac or non-cardiac related. MI was confirmed according to the criteria of “the third universal definition of myocardial infarction”.[11] TVR referred to any percutaneous or surgical revascularization of the previously treated vessel. Stroke was diagnosed by CT scan. Bleeding was classified using the “Bleeding Academic Research Consortium (BARC)” definition, [12] in which BARC ≥3 grade bleeding was considered as major bleeding. The follow-up checkups were performed by telephone after six months and one year after patients’ discharge. Death was confirmed upon issuance of a death certificate from the local police office, whereas MI and TVR were confirmed by a certificate of diagnosis from the hospital where the patient was treated.

### Statistical analysis

Continuous variables were shown as mean value ± standard deviation and were compared with Student’s *t* test if we assume normal random distribution. Variables that were not normally distributed were shown as medians and quartile ranges and compared using the Mann–Whitney U test. Categorical variables were shown as frequencies and compared using the Chi-square test or Fisher’s exact test. Cumulative incidence of one-year MACE was estimated by the Kaplan–Meier curves and assessed using log-rank test. Multivariable regression analysis was performed to identify the independent effect of TRA vs. TFA on in-hospital NACE and one-year MACE. Variables included in the model were as follows: gender, age, prior MI, prior PCI, prior coronary artery bypass surgery, prior stroke, diabetes mellitus, hypertension, hyperlipidemia, diagnosis of acute coronary syndrome, left ventricle ejection fraction (LVEF), use of GP IIb/IIIa inhibitor, size of sheath, three-vessel disease, left main disease, type C lesion, pre-procedure thrombolysis in MI (TIMI) flow, use of drug-eluting stent, and operators with different PCI quantities.

Because the route selection was not randomized, a 1:1 match propensity score analysis was performed to minimize bias. Patients in the TRA group were arranged in order and matched to the most relevant patients in the TFA group by estimating propensity score. Logistic regression analysis was performed to calculate the probability of assignment, and propensity score was calculated from the logistic regression coefficients. Variables included in the logistic model were as follows: gender, age, prior MI, prior PCI, prior coronary artery bypass surgery, prior stroke, diabetes mellitus, hypertension, hyperlipidemia, clinical diagnosis, LVEF, use of GP IIb/IIIa inhibitor, three-vessel disease, left main disease, post-procedure TIMI flows, use of drug-eluting stent, and operators with different PCI quantities. A difference of ≤ 0.01 in the estimated propensity score between TRA and TFA indicated that the two patients were characteristically even and were paired together. The discrimination and precision of the propensity score model was evaluated by the area under the receiver operating curve (ROC) in the logistic regression model and the Hosmer–Lemeshow test.

Among the propensity score-matched patients, a separate regression analysis was performed. Independent analyses were also performed within six pre-specified subgroups. All analyses were conducted using SAS software, version 9.13 (SAS Institute, Cary, North Carolina), and a two-sided p value of <0.05 was considered for statistical significance.

## Results

### Baseline characteristics

A total of 21,242 patients met the inclusion criteria and were included in the analysis. Patients were analyzed based on intension-to-treat principle. From the total, 18,234 patients (85.8%) had PCI from the radial route, and the rest (14.2%) had TFA PCI. The prevalence of TRA in PCI in our institution is increasing. After the propensity score matching, 1,634 pairs were matched. Logistic model showed good stability and showed no discrimination for the matching (ROC area: 0.695; Hosmer–Lemeshow test, *P* = 0.162). Patients in the TRA and TFA groups were followed up for a median term of 385 d. The follow-up at one year was completed in 17,961 (98.5%) in the TRA group, and 2953 (98.2%) in the TFA group.

Patients’ clinical characteristics are shown in Table 1, and the angiographic results are shown in Table 2. Significant differences were detected between TRA and TFA in terms of patients’ medical history and severity of atherosclerosis. After propensity score matching, patients included in the TRA and TFA groups had comparable baseline characteristics. However, patients in the TFA group had more lesions treated than those in the TRA group. The size of sheath of more than 7 French was used in 10.3% in the TFA group and 1.8% in the TRA group (*P* < 0.001), and IABP was more often used in the TFA group (*P* < 0.001). Catheter-related dissection (1.3% vs. 1.2%, *P* = 0.931) and thrombosis (0.3% vs. 0.4%, *P* = 0.897) were similar between the TFA and the TRA group. Contrast volume was higher (152.70 ± 76.79 ml vs. 163.72 ± 95.21 ml, *P* < 0.001), and the total procedure time was longer (38.59 ± 20.12 min vs. 42.73 ± 25.67 min, *P* < 0.001) in the TFA group.

**Table 1:** Baseline characteristics.

Variable	All Patients			Propensity score-matched patients		
	TRA(N = 18234)	TFA(N = 3008)	*P*	TRA(N = 1634)	TFA(N = 1634)	*P*
Age	57.55 ± 10.18	60.15 ± 10.88	<0.001	59.09 ± 10.62	59.91 ± 10.94	0.031
Male	14498 (79.5%)	2096 (69.7%)	<0.001	1125 (68.8%)	1085 (66.4%)	0.135
Prior MI	4145 (22.7%)	842 (28.0%)	<0.001	446 (27.3%)	439 (26.9%)	0.783
Prior CABG	97 (0.5%)	360 (12.0%)	<0.001	57 (3.5%)	57 (3.5%)	1.000
Prior PCI	2631 (14.4%)	619 (20.6%)	<0.001	325 (19.9%)	332 (20.3%)	0.760
Prior stroke	723 (4.0%)	148 (4.9%)	0.017	70 (4.3%)	76 (4.7%)	0.611
Diabetes	4410 (24.2%)	723 (24.0%)	0.859	370 (22.6%)	391 (23.9%)	0.385
Hypertension	10555 (57.9%)	1810 (60.2%)	0.018	962 (58.9%)	983 (60.2%)	0.454
Hyperlipidemia	9773 (53.6%)	1586 (52.7%)	0.270	832 (50.9%)	851 (52.1%)	0.506
PCI indications:			0.017			0.638
STEMI	2664 (14.6%)	391 (13.0%)		256 (15.7%)	237 (14.5%)	
NSTEMI	1123 (6.2%)	202 (6.7%)		118 (7.2%)	113 (6.9%)	
Unstable angina	8239 (45.2%)	1440 (47.9%)		750 (45.9%)	794 (48.6%)	
Stable angina	5381 (29.5%)	842 (28.0%)		440 (26.9%)	425 (26.0%)	
Other	827 (4.5%)	133 (4.4%)		70 (4.3%)	65 (4.0%)	
LVEF(%)	62.07 ± 7.90	60.82 ± 8.14	<0.001	61.14 ± 8.03	61.13 ± 8.11	0.991
Serum creatine	79.86 ± 20.25	80.88 ± 20.99	0.033	79.10 ± 19.13	79.71 ± 21.07	0.456
Peri-procedrual medication						
GP IIb/IIIa	235 (1.3%)	57 (1.9%)	0.012	27 (1.7%)	34 (2.1%)	0.365
LMWH	14301 (78.4%)	2283 (75.9%)	0.002	1279 (78.3%)	1256 (76.9%)	0.335
Fondaparinux	101 (0.6%)	19 (0.6%)	0.604	16 (1.0%)	11 (0.7%)	0.333
Warfarin	57 (0.3%)	21 (0.7%)	0.003	5 (0.3%)	9 (0.6%)	0.281
Procedure according to doctors’ different experiences			<0.001			0.120
< 150 annually	4039 (23.8%)	633 (26.5%)		430 (26.3%)	444 (27.2%)	
150~300 annually	4362 (25.7%)	747 (31.2%)		476 (29.1%)	518 (31.7%)	
> 300 annually	8570 (50.5%)	1012 (42.3%)		728 (44.6%)	672 (41.1%)	

Data represented as n (%) or mean ± SD.

CABG, coronary artery bypass graft; GP IIb/IIIa, Glycoproterin IIb/IIIa inhibitor; LMWH, low molecular weight heparin; LVEF, left ventricle ejection fraction; MI, myocardial infarction; NSTEMI, non ST-segment elevation myocardial infarction; STEMI, ST-segment elevation myocardial infarction; TFA, transfemoral approach; TRA, transradial approach; PCI, percutenous coronary intervention.

**Table 2:** Angiographic characteristics and procedure outcomes.

Variable	All Patients			Propensity score-matched patients		
	TRA (N = 18234, Lesions = 23035)	TFA (N = 3008, Lesions = 3816)	*P*	TRA (N = 1634, Lesions = 2369)	TFA (N = 1634, Lesions = 2508)	*P*
LM involved disease	50 (0.3%)	12 (0.5%)	0.261	158 (9.7%)	166 (10.2%)	0.640
Three vessels disease	6132 (33.6%)	1087 (36.1%)	0.007	649 (39.7%)	662 (40.5%)	0.643
Treated lesion(per person)			<0.001			0.016
1	11442 (62.8%)	1782 (59.2%)		1043 (63.8%)	969 (59.3%)	
2	5271 (28.9%)	917 (30.5%)		470 (28.8%)	495 (30.3%)	
3	1281 (7.0%)	265 (8.8%)		100 (6.1%)	144 (8.8%)	
≥ 4	240 (1.3%)	44 (1.5%)		21 (1.3%)	26 (1.5%)	
Type C lesion*	11352 (49.5%)	2021 (53.2%)	<0.001	1199 (50.8%)	1302 (52.1%)	0.366
Treated CTO lesion*	776 (3.4%)	185 (4.8%)	<0.001	74 (3.1%)	135 (5.4%)	<0.001
Treated ostium lesion*	2895 (12.7%)	734 (19.3%)	<0.001	313 (13.3%)	419 (16.8%)	<0.001
Treated bifurcation lesion*	7670 (33.6%)	1343 (35.4%)	0.041	815 (34.6%)	848 (34.0%)	0.634
Pre-procedure TIMI 3*	16922 (73.9%)	2781 (73.3%)	0.456	1725 (73.3%)	1815 (72.8%)	0.692
Post-procedure TIMI 3*	22500 (98.7%)	3716 (98.3%)	0.056	2318 (98.3%)	2459 (98.4%)	0.629
Sheath size≤6	17908 (98.2%)	2543 (84.5%)	<0.001	1605 (98.2%)	1466 (89.7%)	<0.001
Drug eluting stent	18148 (99.5%)	2971 (98.8%)	<0.001	1610 (98.5%)	1612 (98.7%)	0.766
Stent number (per person)			<0.001			0.005
1	8220 (45.1%)	1226 (40.8%)		739 (45.2%)	666 (40.8%)	
2	5777 (31.7%)	973 (32.3%)		517 (31.6%)	531 (32.5%)	
3	2907 (15.9%)	503 (16.7%)		259 (15.9%)	268 (16.4%)	
4	957 (5.2%)	220 (7.3%)		74 (4.5%)	119 (7.3%)	
≥ 5	373 (2.0%)	86 (2.9%)		45 (2.7%)	50 (3.0%)	
Stent length (mm)	23.64 ± 6.55	23.58 ± 6.75	0.626	23.06 ± 6.88	22.94 ± 6.91	0.467
Stent diameter (mm)	3.06 ± 0.79	3.05 ± 0.85	0.382	3.13 ± 0.46	3.06 ± 0.99	0.280
IABP support	76 (0.4%)	79 (2.7%)	<0.001	8 (0.5%)	36 (2.3%)	<0.001
Contrast volume (ml)	150.66 ± 75.38	161.73 ± 91.94	<0.001	152.70 ± 76.79	163.72 ±95.21	<0.001
Total procedure time (min)	38.75 ± 24.20	43.99 ± 26.52	<0.001	38.59 ±20.12	42.73 ±25.67	<0.001
Intervention complications						
Dissection	170 (0.7%)	49 (1.3%)	0.001	30 (1.3%)	31 (1.2%)	0.931
Thrombosis	36 (0.2%)	17 (0.4%)	0.001	8 (0.3%)	9 (0.4%)	0.897

Data represented as proportion, mean ±SD or median (25^th^ quartile, 75^th^ quartile).

*Compared in the lesion level.

CTO, chronic total occlusion; LM, left main branch; IABP, intra-aortic balloon pump; TFA, transfemoral approach; TIMI, Thrombolysis in myocardial infarction; TRA, transradial approach.

### Major outcomes

**Fig 2:**
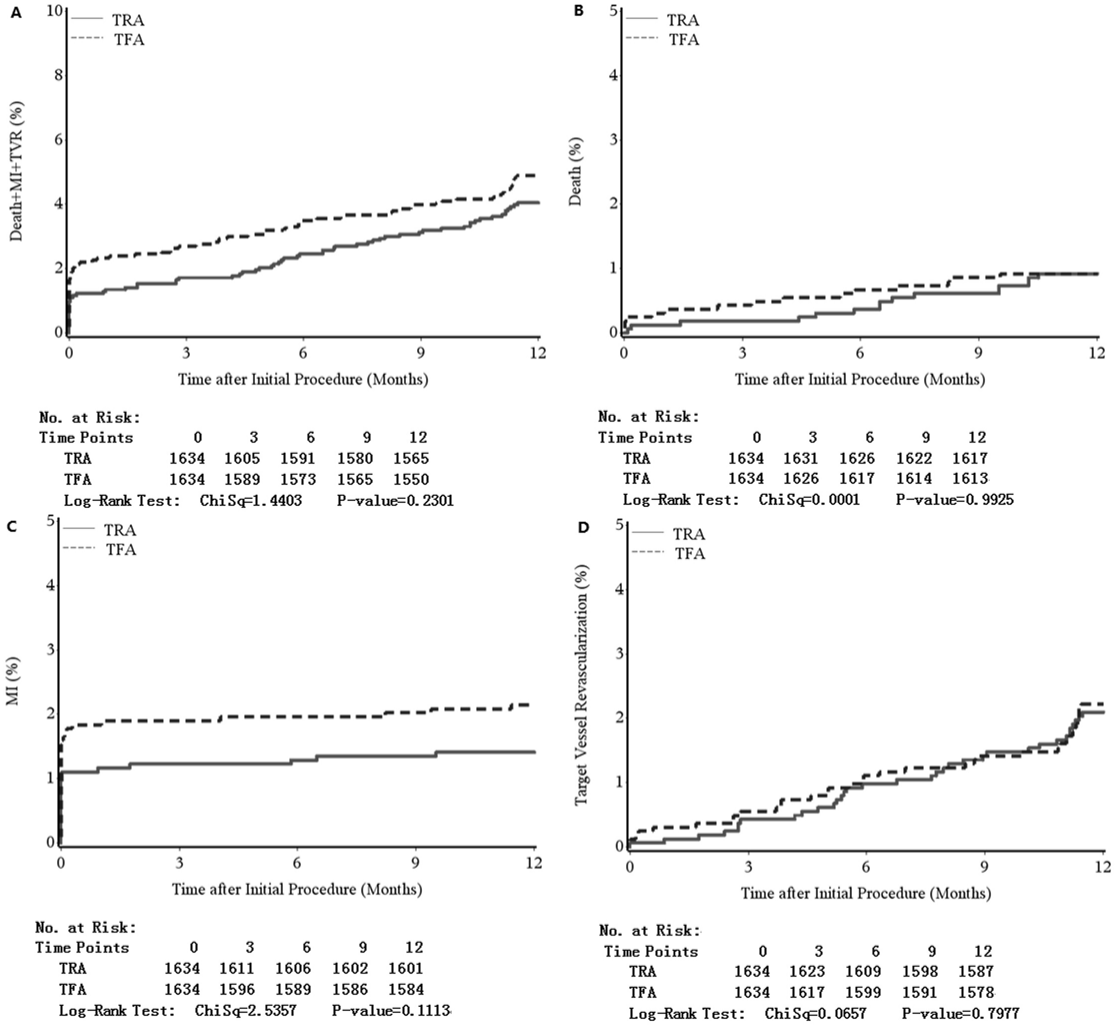
Kaplan-Meier curves for the outcomes between transradial and transfemoral groups of 12 months follow-up in the propensity score-matched patients. (A) Kaplan-Meier curves for occurrence of death between transradial and transfemoral groups of 12 months follow-up in the propensity score-matched patients. (B) Kaplan-Meier curves for occurrence of myocardial infarction between transradial and transfemoral groups of 12 months follow-up in the propensity score-matched patients. (C) Kaplan-Meier curves for occurrence of target vessel revascularization between transradial and transfemoral groups of 12 months follow-up in the propensity score-matched patients. (D) Kaplan-Meier curves for occurrence of major adverse cardiovascular event between transradial and transfemoral groups of 12 months follow-up in the propensity score-matched patients. MI, myocardial infarction; TFA, transfemoral approach; TRA, transradial approach; TVR, target vessel revascularization.

Major outcomes for patients in the TRA and TFA groups are shown in Table 3. The incidence rates of in-hospital NACE (1.8% vs. 4.0%, *P* < 0.001) and one-year MACE (3.9% vs. 5.2%, *P* = 0.001) were higher in the TFA group than in the TRA group. However, in the propensity score-matched patients, the rate of in-hospital NACE (1.8% vs. 3.9%, *P* < 0.001) was higher in the TFA group, and the rate of one-year MACE was similar between TRA and TFA groups (4.7% vs. 4.9%, *P* = 0.272). The higher rate of NACE in the TFA group was mainly due to the higher rate of major bleeding (0.6% vs. 1.8%, *P* < 0.001) and the increased rate of MI (1.1% vs. 1.9%, *P* = 0.060). Meanwhile, in-hospital composite endpoints, including death, MI, and stroke, occurred more in the TRA group (1.2% vs. 2.3%, *P* = 0.016). The Kaplan–Meier curves of one-year major outcomes are shown in Fig 2. No statistical difference was detected in MACE or each component of MACE between the TRA and TFA groups in the log-rank test. The total hospital and the post-procedure stays were both longer in the TFA group than in the TRA group (*P* < 0.001).

**Table 3:** Major outcomes.

	All patients			Propensity score-matched patients		
Variable	TRA(N = 18234)	TFA(N = 3008)	*P*	TRA(N = 1634)	TFA(N = 1634)	*P*
**In-hospital outcomes**						
NACE	336 (1.8%)	119 (4.0%)	<0.001	29 (1.8%)	63 (3.9%)	<0.001
All-cause death	14 (0.1%)	10 (0.3%)	<0.001	1 (0.1%)	5 (0.3%)	0.218
Cardiac death	12 (0.1%)	10 (0.3%)	<0.001	1 (0.1%)	5 (0.3%)	0.218
MI	227 (1.2%)	55 (1.8%)	0.013	18 (1.1%)	31 (1.9%)	0.060
TVR	24 (0.1%)	6 (0.2%)	0.427	1 (0.1%)	3 (0.2%)	0.625
Major bleeding	94 (0.5%)	58 (1.9%)	<0.001	9 (0.6%)	29 (1.8%)	<0.001
Stroke	9 (0.0%)	3 (0.1%)	0.236	1 (0.1%)	3 (0.2%)	0.625
Death, MI and Stroke	246 (1.3%)	66 (2.2%)	<0.001	20 (1.2%)	38 (2.3%)	0.016
Total hospital stay (d)	6 [4,7]	7 [5,10]	<0.001	6 [4,7]	6 [5,9]	<0.001
Post-procedure stay (d)	3 [2,4]	4 [3,5]	<0.001	3 [2,4]	3 [3,5]	<0.001
**1 year outcomes**						
MACE	701 (3.9%)	154 (5.2%)	0.001	67 (4.1%)	80 (4.9%)	0.272
All-cause death	102 (0.6%)	28 (0.9%)	0.022	15 (0.9%)	15 (0.9%)	1.000
Cardiac death	53 (0.3%)	20 (0.7%)	0.003	7 (0.4%)	12 (0.7%)	0.247
MI	262 (1.5%)	67 (2.3%)	0.002	23 (1.4%)	35 (2.1%)	0.111
TVR	382 (2.1%)	70 (2.4%)	0.383	35 (2.1%)	36 (2.2%)	0.905

Data represented as n (%) or median (25th quartile, 75th quartile).

MACE, major adverse clinical event; MI, myocardial infarction; NACE, net adverse clinical events; TVR, target vessel revascularization; TFA, transfemoral approach; TRA, transradial approach.

The adjusted rates of in-hospital NACE was lower in the TRA group [odds ratio (OR), 0.53; 95% CI from 0.40 to 0.71], and the adjusted rate of one-year MACE were similar between TRA and TFA [hazards ratio (HR), 1.01; 95% confidence interval (CI), from 0.96 to 1.06]. In the propensity score-matched patients, the adjusted rate of in-hospital NACE was still lower in the TRA group (OR, 0.46; 95% CI, from 0.29 to 0.73), and the adjusted rate of one-year MACE remained similar between TRA and TFA (HR, 1.01; 95% CI, from 0.94 to 1.08) (Table 4).

**Table 4:** The adjusted rates for the major endpoint of TRA vs. TFA.

	All Patients		Propensity score-matched patients	
Outcomes	OR/HR (95% CI)	*P*	OR/HR (95% CI)	*P*
In-hospital NACE	0.53 (0.40, 0.71)	<0.001	0.46 (0.30, 0.73)	0.001
One year MACE	1.01 (0.96, 1.06)	0.786	1.01 (0.94, 1.08)	0.779

CI, confidence interval; HR, hazard ratio; MACE, major adverse cardiovascular events; NACE, net adverse clinical events; OR, odds ratio; TFA, transfemoral approach; TRA,transradial approach.

### Access complications and major bleeding

Access site complications and peri-procedure bleeding occurred more in the TFA group (*P* < 0.05) than in the TRA group. In the propensity score-matched patients, TRA was associated with lower rates of access site complications (1.5% vs. 4.7%, *P* < 0.001) and access site-related major bleeding (0.4% vs. 1.2%, *P* = 0.005). However, no difference was detected with regard to non-access site-related major bleeding (0.2% vs. 0.6%, *P* = 0.076) (Table 5).

**Table 5:** Access site complications and bleeding.

Variable	All patients			Propensity score-matched patients		
	TRA(N = 18234)	TFA(N = 3008)	*P*	TRA(N = 1634)	TFA(N = 1634)	*P*
**Access site complications**	220 (1.2%)	145 (4.8%)	<0.001	25 (1.5%)	77 (4.7%)	<0.001
Hematoma	211 (1.2%)	118 (3.9%)	<0.001	22 (1.3%)	65 (4.0%)	<0.001
Aneurysm	1 (0.0%)	13 (0.4%)	<0.001	0 (0.0%)	3 (0.2%)	0.250
Arteriovenus fistula	1 (0.0%)	9 (0.3%)	<0.001	0 (0.0%)	6 (0.4%)	0.031
Retroperiton-eal hematoma	0 (0.0%)	6 (0.2%)	<0.001	0 (0.0%)	2 (0.1%)	0.500
**In-hospital bleeding**						
Access major bleeding	64 (0.4%)	44 (1.5%)	<0.001	6 (0.4%)	20 (1.2%)	0.005
Non access major bleeding	30 (0.2%)	14 (0.5%)	0.003	3 (0.2%)	9 (0.6%)	0.076
BARC ≥ 2 grade bleeding	1035 (5.7%)	410 (13.6%)	<0.001	97 (5.9%)	224 (13.7%)	<0.001

Data represented as n (%).

BARC, Bleeding Academic Research Consortium; TFA, transfemoral approach; TRA, transradial approach.

### Subgroup analyses

The adjusted rates of in-hospital NACE and one-year MACE in the subgroup patients are shown in S1 Fig and S2 Fig Consistent with the results from the entire patient population, patients in the TRA group had better NACE, but similar MACE at one-year follow-up compared with TFA in most subgroups. No significant interaction was detected between the radial route and the subgroup characteristics.

## Discussion

This study compared the outcomes between different route selections in an extremely high-volume PCI institution. Compared with patients in the TFA group, patients in the TRA group had a lower rate of in-hospital NACE, which was mainly due to lower rates of MI and major bleeding.

The annual PCI volume of the institution was shown to be a predictor for the TRA outcomes.[10] Because TRA is a skill-demand technique, and centers with high volume PCI are more willing to perform new strategies. TRA implementation is more prevalent in large heart centers. We selected one high-volume PCI center, and results showed that TRA was much safer compared with TFA based on in-hospital outcomes, including lower rates of NACE, major bleeding, and composite endpoints of death, MI, and stroke. This finding was in agreement with the results of previous studies performed in different populations. The RIVAL study showed a lower rate of in-hospital NACE (including death, MI, stroke, and ACUITY bleeding) in acute coronary syndrome patients when TRA was used instead of TFA.[10] The PREVAIL study showed similar rate of short-term death, but lower rate of MI in TRA patients among unselected PCIs.[13] Similarly, we found a decreased rate of MI (1.1% vs. 1.9%, *P* = 0.060) with TRA instead of TFA, even though it has not reached significance. The underlying reason for the decreased MI in the TRA group is unclear. However, the relatively larger sheath and guiding catheters in the TFA group could result in more PCI-related MIs than that in the TRA group.

The medium-term outcomes between TRA and TFA groups varied across studies. Our study was performed in a stable population undergoing elective PCI, and showed similar rates for one-year MACE between the TRA and TFA groups. The CREDO–Kyoto study showed similar three-year death rates between TRA and TFA in a non-acute MI population.[14] Meanwhile, propensity score-matched Italian cohort with acute MI patients showed lower two-year death rates with TRA than with TFA.[15] Randomized trial results suggested that high-risk patients showed lower death rates with TRA than with TFA.[16–18] However, this finding was not detected in any subgroup analysis in the present study.

RIVAL study found lower ACUITY bleeding rate in the TRA group,[10] which was attributed to low rates of access site-related bleeding. Access site-related bleeding accounted for 50% to 70% of all bleeding incidents in patients who underwent PCI.[19] Thus, using TRA instead of TFA could be a good strategy for reducing bleeding events, particularly access site-related bleeding. Access site complications occurred more frequently and were more severe in the TFA group than in the TRA group. The incidence rate of hematomas, which prolonged hospital stay, nearly tripled in the TFA group compared with the TRA group. BARC 2 bleeding (excessive bleeding), which was closely related to access site complications, also occurred more frequently in the TFA group than in the TRA group. Severe complications, such as pseudoaneurysm, arterio-venous fistula, retroperitoneal hematoma are rarely occurred in the TRA group. By contrast, these were frequent complications in the TFA group. Intrinsic anatomical differences exist between the radial and the femoral arteries, thus interventionists can simply accomplish puncturing and easily achieve hemostasis with TRA instead of TFA.[20] In addition, inadequate compression of the femoral artery is the most common reason for the large hematomas.[21]

Our study has the following limitations. First, patient assignment was not randomized, but was based on doctor’s preference. This could lead to uneven baseline characteristics. However, we used the propensity score to adjust for the disparities. Whereas hidden confounders could not be completely removed, and thus, large randomized trials are required. Second, because of the retrospective extraction, we failed to obtain the crossover rate when one route for the catheterization failed. In analyzing the route effect on major outcomes, we used the intention-to-treat principle in dividing patients into groups, which was consistent with the statistical strategy used in previous observational studies.[21] Third, this study was performed in a single heart center with high-volume PCI. Thus, the effect of TRA in high volume PCI centers may be well-represented. However, we failed to show the effect of TRA in relatively low or median volume PCI-capable hospitals. Besides, this study included patients from 2006 to 2011, while only 85% of patients had TRA PCI. Nowadays, in Europe at least, centers with high expertise in TRA perform more than 95% of cases by TRA, so the present result might be used as reference in those countries, and the results of those centers are also expected.

## Conclusions

Compared with TFA, TRA is much safer and results in lower rate of NACE during hospitalization in patients undergoing elective PCI. We suggest that TRA should be routinely adopted in PCI-capable hospitals.

## Supporting Information

S1 Fig Forest plot of prespecified subgroup analyses of in-hospital net adverse clinical events. (TIF)

S2 Fig Forest plot of prespecified subgroup analyses of one-year major adverse cardiovascular events. CI, confidence interval; HR, hazard ratio; LVEF, left ventricle ejection fraction; NSTEMI, non ST-segment elevation myocardial infarction; SAP, stable angina pectoris; STEMI, ST-segment elevation myocardial infarction; UAP, unstable angina pectoris.
(TIF)
