# Supplementary figures and images for: Comparison of Short- and Medium-Term Clinical Outcomes between Transradial Approach and Transfemoral Approach in a High-Volume PCI Heart Center in China
Source: PLoS One. 2015 Mar 31;10(3):e0118491. doi: 10.1371/journal.pone.0118491 (PMC4380354; doi:10.1371/journal.pone.0118491)

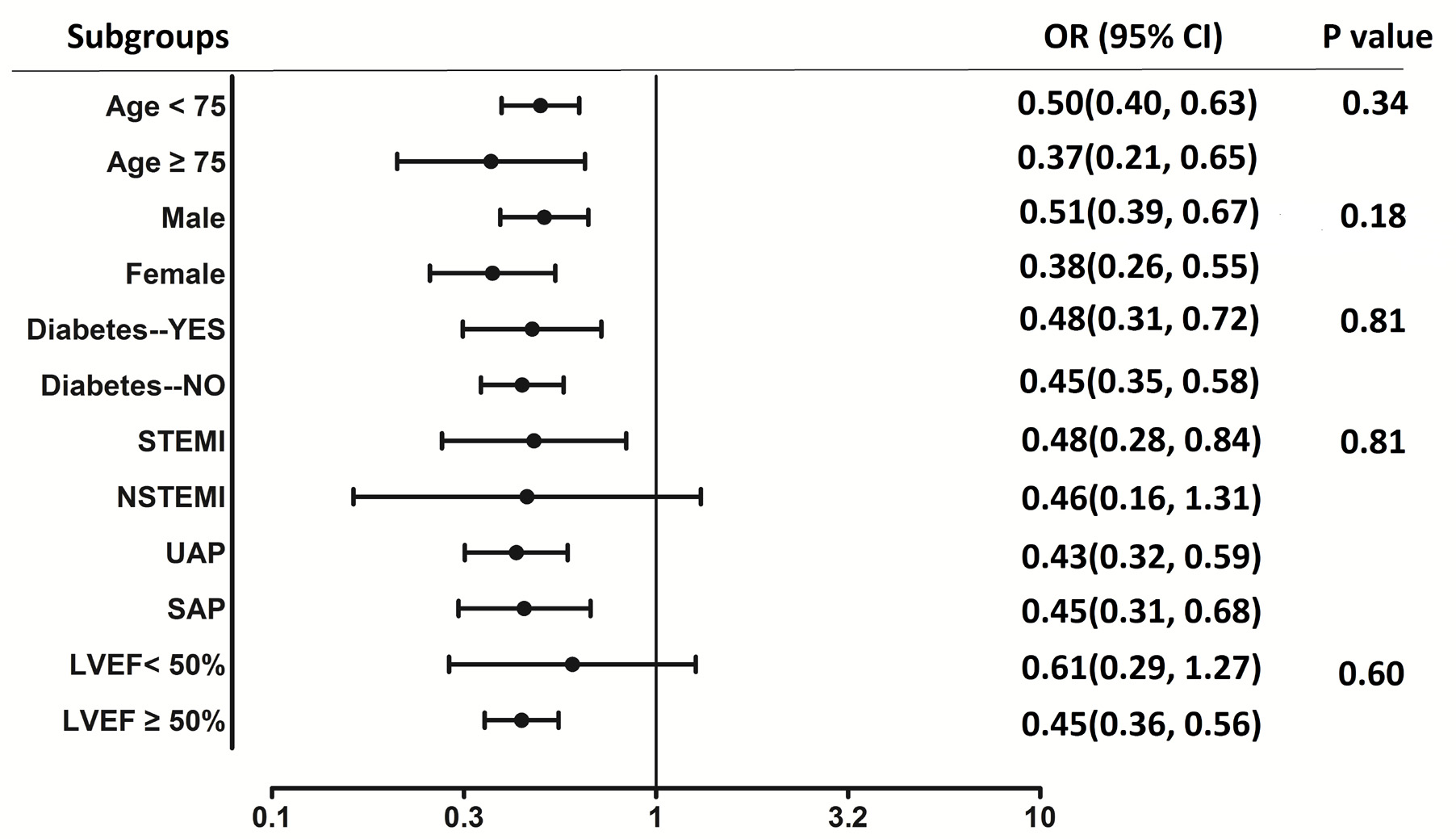

Supplement: S1 Fig — (TIF) [file pone.0118491.s001.tif]

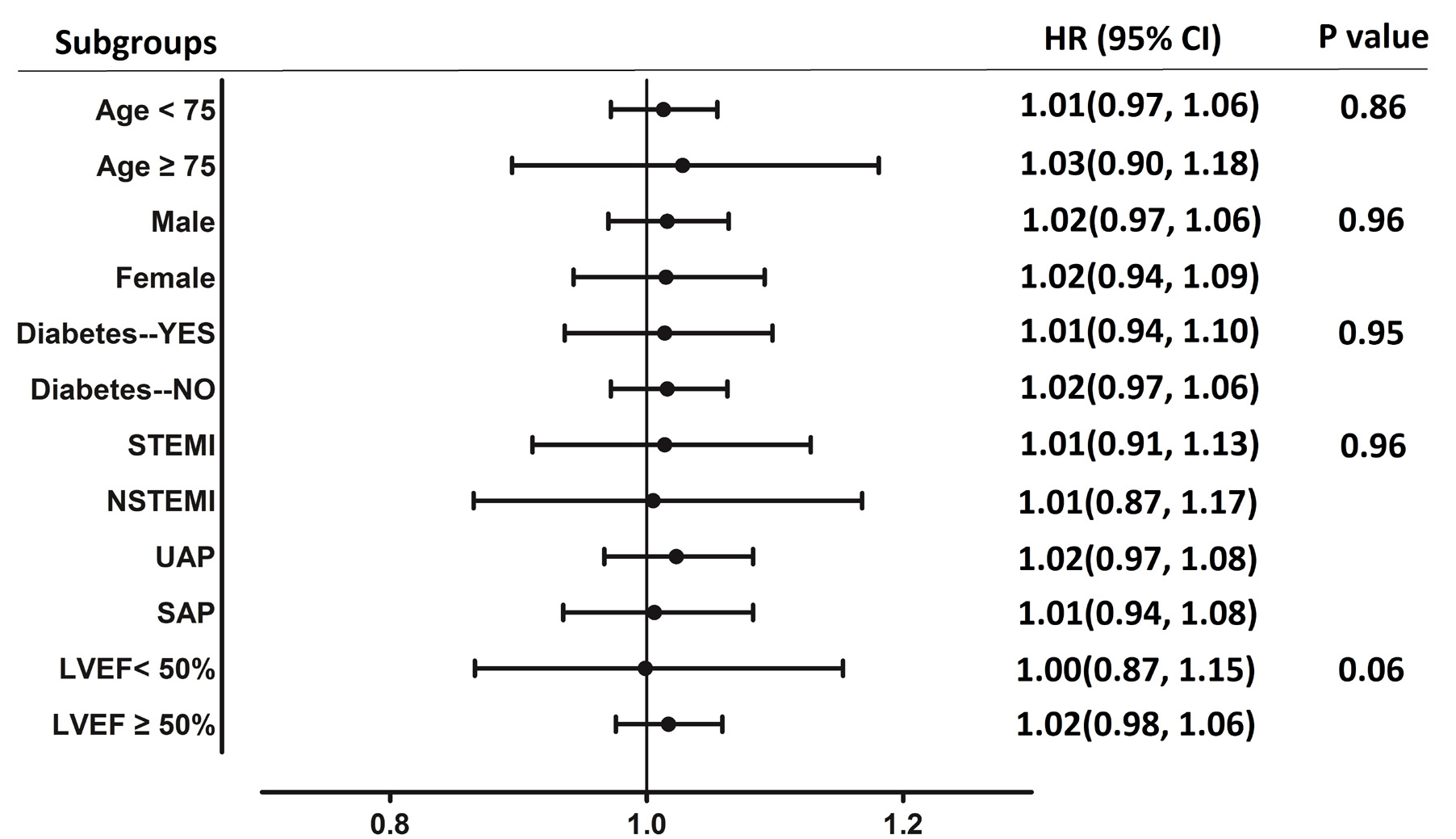

Supplement: S2 Fig — CI, confidence interval; HR, hazard ratio; LVEF, left ventricle ejection fraction; NSTEMI, non ST-segment elevation myocardial infarction; SAP, stable angina pectoris; STEMI, ST-segment elevation myocardial infarction; UAP, unstable angina pectoris. (TIF) [file pone.0118491.s002.tif]
